# The Effects of Replacing Soybean Meal with Rapeseed Meal, Cottonseed Cake, and Fava Beans on the Milk Yield and Quality Traits in Milking Ewes

**DOI:** 10.3390/ani12030274

**Published:** 2022-01-22

**Authors:** Aphrodite I. Kalogianni, Marios Moschovas, Foteini Chrysanthakopoulou, Thomai Lazou, Georgios Theodorou, Ioannis Politis, Ioannis Bossis, Athanasios I. Gelasakis

**Affiliations:** 1 Laboratory of Anatomy and Physiology of Farm Animals, Department of Animal Science, School of Animal Biosciences, Agricultural University of Athens (AUA), Iera Odos 75 Str., 11855 Athens, Greece; afrokalo@aua.gr (A.I.K.); moschovas@aua.gr (M.M.); 2 Agricultural Cooperative ‘Agrinio Union’, Papaioannou 24 Str., 30131 Agrinio, Greece; chrysanthakopoulouf@gmail.com; 3 Laboratory of Hygiene of Foods of Animal Origin—Veterinary Public Health, School of Veterinary Medicine, Faculty of Health Sciences, Aristotle University of Thessaloniki, 54124 Thessaloniki, Greece; tlazou@vet.auth.gr; 4 Laboratory of Animal Husbandry, Department of Animal Science, School of Animal Biosciences, Agricultural University of Athens (AUA), Iera Odos 75 Str., 11855 Athens, Greece; gtheod@aua.gr (G.T.); i.politis@aua.gr (I.P.); 5 Laboratory of Animal Husbandry, Department of Agricultural Sciences, School of Agriculture, Forestry and Natural Resources, Aristotle University of Thessaloniki (AUTH), 54124 Thessaloniki, Greece; bossisi@agro.auth.gr

**Keywords:** soybean meal, fava beans, rapeseed meal, cottonseed cake, dairy sheep, milk yield, milk quality

## Abstract

**Simple Summary:**

The substitution of soybean meal in farm animal diets is considered vital for the economic and environmental sustainability of the livestock sector. However, data regarding the effects of a soybean meal replacement on the milk yield and quality traits in dairy sheep are scarce. In our study, two isonitrogenous and isoenergetic diets were used, with soybean meal of a typical ration being replaced by a mixture of rapeseed meal, cottonseed cake, and fava beans. The milk yield and the body condition scores were recorded, and milk samples were analyzed monthly for their fat, protein, lactose, and total solids yields, as well as for somatic cell counts, total bacterial counts, pH, electrical conductivity, and the refractive index. Daily and 100-day fat yields were significantly increased in the group fed the experimental ration and the electrical conductivity was significantly decreased in the same group, while no adverse effects on any of the rest of the studied milk production traits were observed.

**Abstract:**

The replacement of soybean meal (SBM) from intensively reared dairy sheep diets has emerged as a significant challenge for sustainable production. However, the effects of this replacement on milk production have not been sufficiently elucidated. The objective of this study was to prospectively assess the effects of replacing SBM with a mixture of alternative protein sources on the milk yield (MY) and the milk quality traits (MQT) in intensively reared dairy sheep. A total of 112 multiparous, purebred milking ewes of the Chios and Frizarta breeds, from two intensive dairy sheep farms, were involved in the study, postweaning, and were assigned to either the control (CR) or the experimental ration (ER) group. In the ER, 3/4 of the SBM was replaced by a mixture of rapeseed meal, cottonseed cake, and fava beans, producing a ration of a similar nutritional value. MY, MQT, and body condition scores were recorded for each individual ewe monthly for a period of 4 months during lactation. The experimental ration was associated with beneficial effects on daily and 100-day fat yields and on the electrical conductivity of milk as an improved udder health status indicator, with no adverse effects on any of the rest of the studied milk production traits.

## 1. Introduction

The demand for sheep milk and products thereof (e.g., cheese, yoghurt, and butter) has increased over the years due to their perceived high nutritional value and the consumer demands to produce niche and premium-quality dairy products [1]. This demand-driven evolution of the sheep milk processing sector has dragged the tendency towards the intensification of production and the modernization of husbandry systems, mainly in the developed world, as exemplified by European countries in the Mediterranean basin (i.e., Greece, Italy, France, and Spain). Among the factors affecting the sustainability of these systems, evidence-based nutrition and precision feeding remain the cornerstones supporting the sufficient exploitation of highly productive dairy sheep breeds [2] in regard to their milk (i) quantity and quality traits (e.g., milk yield, protein, fat, lactose, total solids, fatty acid profile, etc.), (ii) technological and coagulation properties, and (iii) organoleptic traits.

In general, dairy ewe nutrition is characterized by increased demands in energy and protein during the milking period. Particularly in intensive farms, nutrients are supplied inside the barn by feeding concentrates of high nutritional value, gradually transforming grazing-oriented traditional sheep farming systems to zero-grazing indoor systems [3]. Soybean meal (SBM) currently constitutes the most widely used protein-rich feedstuff in the livestock sector for meat and milk production. It is the co-product of soybean oil extraction and represents approximately 70% of the consumed oilseed meals globally [4]. It is highly preferred in diets of dairy ruminants due to its high crude protein (CP) content (44–56% of dry matter (DM)) and nitrogen digestibility (about 80%); it also contains crude fiber (CF) ca. 1.5–6.0% of DM, fat ca. 2% of DM, and 2.0 Mcal/kg net energy for lactation (NEL) [5]. Despite the unquestionable feeding value of SBM, its partial or total substitution in farm animal diets has emerged as an imperative need due to logistic, economic, and environmental burdens. The USA, Brazil, and Argentina rank first in the list of the SBM producing and exporting countries, continuously intensifying their production despite the recognized environmental impacts imposed by its cultivation [6]. Interestingly, more than 40% of the global available SBM is exported to the EU due to the negligible self-sufficiency of the latter, via an economically and environmentally detrimental transatlantic trading system [7]. In addition, soybean is the most widely used genetically modified crop, opposing the consumer awareness of genetically modified organisms [8].

A combination of grain legumes and the by-products of oil plants are a promising alternative protein source in ruminant nutrition due to (i) their high nutritional value, (ii) the improvement of soil fertility and the reduction of nitrogenous fertilization induced by legume cultivation, (iii) their potential cultivation in less fertile, non-irrigated fields, (iv) the exploitation of industrial by-products within the circular agricultural economy model, and (v) the lack of competition with human nutrition [7,9]. The grain legumes of *Leguminosae* family (e.g., fava bean, pea, lupin) and oil plants (e.g., rape and cotton) have been studied as alternative feed resources in both monogastric and ruminant farm animals, though they display contradictory effects on their productivity [10,11,12,13,14,15,16,17,18]. In Greece, three popular, locally produced feedstuffs integrated into farm animal diets as protein sources are rapeseed meal, cottonseed cake, and fava beans. Rapeseed (*Brassica* spp.) constitutes a relatively new cultivation which has emerged mainly during the last decade in the country and has been exploited for the production of biodiesel and for its soil fertilizing capacity. Rapeseed meal is extensively used with meat and wool sheep as an efficient alternative to SBM, offering similar energy, digestibility, and protein degradability comparable to SBM [19]. Rapeseed meal is rich in protein (CP ca. 33–45% of DM) and fiber (CF ca. 9–18% of DM) content, and contains ca. 2% fat and 1.70 Mcal/kg NEL [20]. Cotton (*Gossypium* spp.) is a customary and extensively cultivated crop with a long tradition and experience in its cultivation in many parts of the country. Cottonseed cake, a by-product of the textile industry, is a valuable feedstuff for ruminants given its high protein content (CP ca. 20–50% of DM) and its resistance to gossypol toxicity, in contrast to monogastric animals [21]. Cottonseed cake also contains CF ca. 7–17% of DM, fat ca. 2–10%, and ca 2.0 Mcal/kg NEL [16,22]. Fava bean crop (*Vicia faba* L. minor) is suitable for cultivation in unfavored soils under less intensive and/or organic production systems, given its limited water and fertilizer demands and the consequent low environmental footprint. It is abundant in protein (CP ca. 25–35% of DM) and it contains CF ca. 9–11% of DM, fat ca. 2% of DM, and 1.70 Mcal/kg NEL [23,24]. Although its high ruminal nitrogen degradability and the presence of antinutritional factors (tannins and pyrimidine glycosides) have hindered its preference in intensive farming systems [25,26], the currently available low levels of tannins or tannin-free cultivars, and the implementation of technological treatments such as the extrusion, have improved its nutritional value and enhanced its potential use in ruminant diets [26,27].

Dairy sheep farming is the most dynamic livestock sector in Greece. In the last two decades, the sector has rapidly evolved to cover the increasing demands for the Protected Designation of Origin feta cheese and sheep yoghurt, with the intensification of farming systems being the driving force. Consequently, zero-grazing, high-input farms have emerged and have increased rapidly in the mainland. This transformation has led to the development of balanced diets and more sophisticated feeding protocols to efficiently meet the nutritional requirements of high-yielding animals within a reasonable production cost. Such diets include SBM as the main protein source, utilized to meet corresponding requirements of ewes during lactation, since the evidence to support its efficient substitution with alternative protein sources in terms of animal performance is lacking.

The hypothesis here was that partial replacement of SBM with other protein-rich grain legumes and by-products of oil plants has no adverse effects on the milk yield and quality. To test this hypothesis, the objective of this study was to prospectively evaluate the effects of the partial replacement of SBM with a mixture of rapeseed meal, cottonseed cake, and fava beans on the milk yield and milk quality traits (fat, protein, lactose, total solids yield, somatic cell count (SCC), total bacterial count (TBC), electrical conductivity (EC), refractive index (RI), and pH) in intensively reared dairy sheep of two indigenous Greek breeds.

## 2. Materials and Methods

### 2.1. Animals and Diets

Two intensive dairy sheep farms located at Aetolia-Acarnania in Western Greece were involved in the study. A total of 112 purebred, multiparous (2nd, 3rd and ≥4th parity), milking ewes at postweaning (50 days post-partum), namely, 64 Frizarta (Farm A) and 48 Chios (Farm B), were randomly selected and enrolled in a 4-month prospective study. Initially, in each farm, the selected ewes were homogeneously allocated into two equal groups (with 32 Frizarta and 24 Chios each, for Farm A and B, respectively) according to their parity number, their daily milk yield (DMY) and the milk quality traits (MQT, i.e., fat, protein, lactose, total solids yield, SCC, TBC, EC, RI, and pH) and were assigned to either the control (C) or the experimental (E) groups. The two groups were permanently housed in separate pens and were mechanically milked twice a day with a 12-h interval (8:00 a.m. and 8:00 p.m.), following the routine of the farm. In both groups, concentrates were fed in a pelleted form, whereas alfalfa hay (18% CP) and wheat straw of a similar nutritional value were equally supplied. The ration fed in the group C ewes was a typical one, incorporating 20% of SBM as the main protein source. In group E, 3/4 of SBM was replaced by a mixture of locally produced rapeseed meal, cottonseed cake, and fava beans to produce a ration of similar nutritional value. The assessment of the nutritional value of the two rations was conducted following standard procedures according to the Association of Official Agricultural Chemists (AOAC) in an accredited laboratory. The composition and the nutritional values of the two rations are presented in Table 1.

During the study, the diets (concentrates and roughages) of the two groups were isocaloric and isonitrogenous and were adapted to meet the nutritional demands of the ewes according to their lactation stage and their milk production level. On the contrary, the fiber content (neutral detergent fiber (NDF), acid detergent fiber (ADF), and acid detergent lignin (ADL)) was lower in the group C ration (CR) compared to the group E ration (ER), while the starch content was higher in CR. The concentrate quantities ranged from 1.0 to 1.3 kg/ewe/day with alfalfa hay from 1.1 to 1.4 kg/ewe/day, regularly tuned to meet the nutrient requirements of the animals during the study. Quantities of concentrates were constantly adjusted to the milk yield of each ewe and the additional demanded dry matter for high yielding animals was individually provided in the milking parlor. In any case, in the two groups, the quantities of concentrates and alfalfa hay were equal for equally producing animals. Concentrates were provided in feeders, both during (in the milking parlor) and after milking (in the barn), twice a day, while roughages were fed twice a day after milking; all feed refusals were removed before the next feeding session.

### 2.2. Milk Sampling and Analyses

Following a 30-day adaptation period of the diets (at 80 days post-partum) the enrolled ewes in the two farms were prospectively studied monthly for 4 months, including the adaptation period. In every sampling occasion, the milk yield was recorded, and the milk samples were collected from each individual ewe and were transferred to the lab for chemical analyses. The milk yield recording and milk sampling were performed using ICAR (International Committee of Animal Recording)-approved equipment (Waikato Milkmeter, InterAg, Hamilton, New Zealand) and protocols during morning milking. Two milk samples (ca. 70 mL each) were collected per animal. The first was used for TBC estimation and was aseptically collected (proper udder and teat cleaning with antiseptic towels, discarding of first milk streams, and collection of composite samples from both half udders), before the milk yield recording. The second was collected from the milkmeter’s sampler and was used for the rest of the analyses. Sodium azide (sodium azide tablets, Supelco^®^, Merck Milipore, Burlington, MA, USA) was added, and the samples were transferred under cool storage conditions (4 °C) and were analyzed within 24 h. Milk samples were analyzed for fat, protein, lactose, total solids contents (MilkoScan^TM^ FT+, Foss, Hilleroed Denmark ), SCC (Fossomatic^TM^ FC, Foss, Hilleroed Denmark), and TBC (Bactoscan^TM^ FC+, Foss, Hilleroed Denmark). Daily milk, fat, protein, lactose, and total solids yields were calculated using the morning milking records and were adjusted following the ICAR methods [28]. The physicochemical characteristics of milk samples, namely the pH, EC, and RI, were measured at 20 °C with a pen-type pHmeter-conductometer (EZDO 7200, GOnDO Electronic Co., LTD, Taipei, Taiwan) and a handheld refractometer (RHB-32ATC, Laxco, Inc., Mill Creek, WA, USA) according to the brix scale, respectively. At the end of the study, the total milk, fat, protein, lactose, and total solids yields were calculated for the 100 days of the experiment using the Fleischmann method and the ICAR recommendations [28]. Moreover, the body condition score (BCS) was recorded by the same veterinarian in each sampling using a five-degree scale (1 = emaciated, 5 = obese) [29].

### 2.3. Statistical Analyses

SPSS v23 software (IBM Corp., Armonk, NY, USA) was used for the statistical analyses, with the statistical significance being set at the 0.05 level. Initially, SCC and TBC were log-transformed, and the Kolmogorov–Smirnov test was used to test for normality. Descriptive statistics (mean ± standard error) were calculated for the milk quality and quantity traits for groups C and E throughout the study. The following mixed linear regression model was formulated for the assessment of the effects of the two diets on DMY and MQT:

Y_ijklm_ = μ + F_i_ + G_j_ + P_k_ + S_l_ + a_1_ × BCS + E_m_ + δ_ml_ + e_ijklm_ (model 1)

where Y_ijklm_ = dependent variables (daily milk, fat, protein, lactose, total solids yield, logarithm of SCC (logSCC), logarithm of TBC (logTBC), EC, pH, and RI); μ = intercept; F_i_ = fixed effect of the farm (i = 2 levels; 0 = Farm A, 1 = Farm B); G_j_ = fixed effect of the ration (j = 2 levels; 0 = control ration, 1 = experimental ration); P_k_ = fixed effect of the parity number (k = 3 levels; 2nd, 3rd, ≥4th parity); S_l_ = fixed effect of the sampling occasion (l = 4 levels; 1st to 4th sampling occasion); a_1_ = fixed effect of the regression coefficient of BCS; E_m_ = random variation of the m^th^ ewe; δ_ml_ = repeated variation of the m^th^ ewe in the lth sampling occasion; e_ijklm_ = residual error.

Akaike’s information criterion (AIC) value was used for the selection of the most appropriate covariance structure in the mixed linear model and the first-order autoregressive was selected as the most appropriate one.

An analysis of covariance was used to assess the effects of the diet on the 100-day milk, fat, protein, lactose, and total solids yields, as described in the following model:

Y_ijk_ = μ + F_i_ + G_j_ + P_k_ + e_ijk_ (model 2)

where Y_ijkl_ = dependent variables (100-d milk, fat, protein, lactose and total solids yield); μ = intercept; F_i_ = fixed effect of the farm (i = 2 levels; 0 = Farm A, 1 = Farm B); G_j_ = fixed effect of the ration (j = 2 levels; 0 = control ration, 1 = experimental ration); P_k_ = fixed effect of the parity number (k = 3 levels; 2nd, 3rd, ≥4th parity); and e = residual error.

Τhe assumptions of normal distribution, homoscedasticity, and linearity for the models were checked by the assessment of a scatterplot of standardized predicted values against the standardized residuals and the probability-probability and quantile-quantile plots of standardized residuals.

## 3. Results

### 3.1. Descriptive Statistics

**Figure 1:**
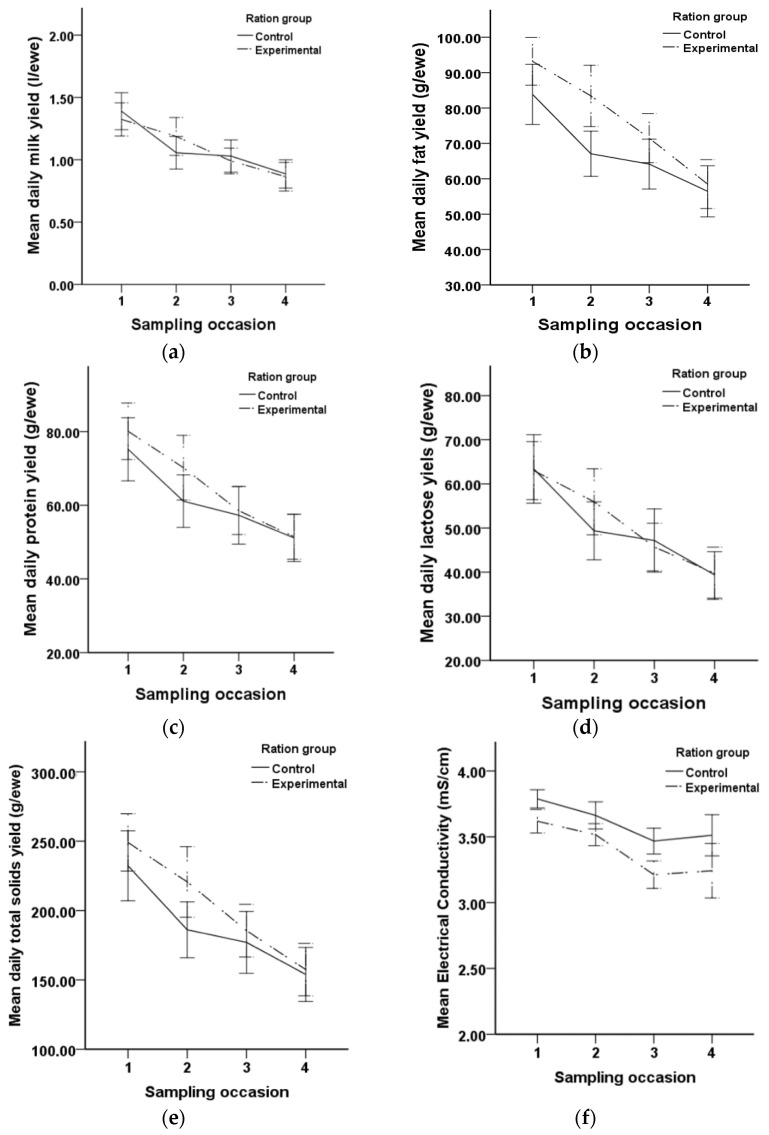

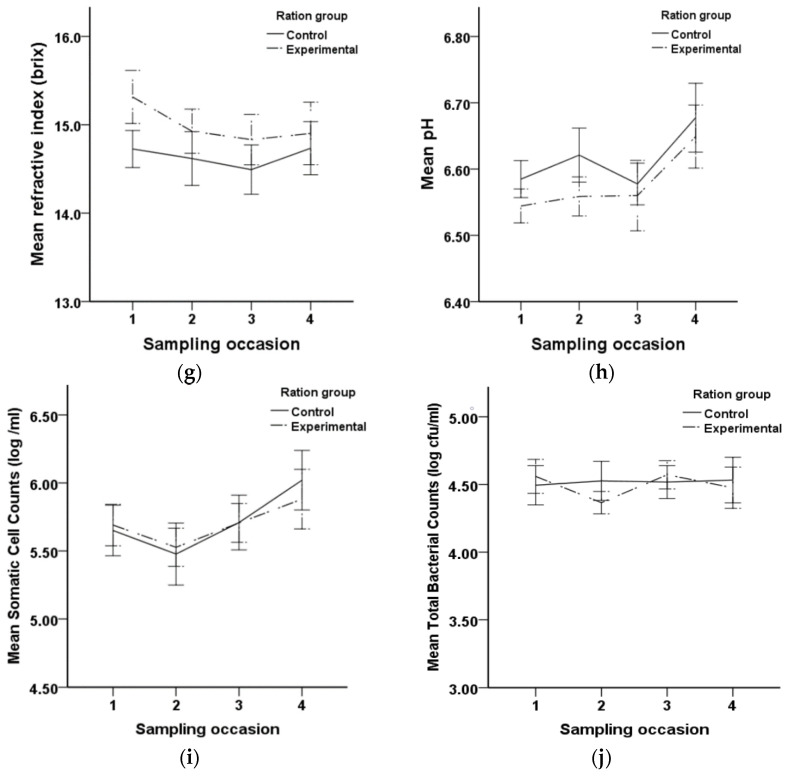
Mean values of (**a**) daily milk yield; (**b**) daily fat yield; (**c**) daily protein yield; (**d**) daily lactose yield; (**e**) daily total solids yield; (**f**) electrical conductivity; (**g**) refractive index; (**h**) pH; (**i**) logarithm of somatic cell counts; and (**j**) logarithm of total bacterial counts, for the two groups during the study.

Figure 1 demonstrates the progress of DMY and the studied MQT for the two groups during the study. The average DMY continuously decreased from the middle to the end of lactation in both groups, ranging from 1.4 to 0.9 l for group C ewes and from 1.3 to 0.9 l for group E ewes. A similar declining trend was observed for the daily fat, protein, lactose, and total solids yields. During the study, the mean daily fat, protein, lactose and total solids yields varied from 83.9 to 56.4 g, 75.2 to 51.1 g, 63.4 to 39.7 g, and 232.3 to 157.5 g in group C, respectively, while in group E the values varied from 93.2 to 58.5 g, 80.1 to 51.5 g, 63.0 to 39.7 g, and 249.1 to 157.5 g, respectively. Table 2 summarizes the mean values of daily and total milk yields and the milk quality traits. The mean values of BCS varied from 2.7 to 2.9 in both groups, following a similar trend during the study.

### 3.2. The Effects of Diet on the Daily and Total Milk Yields and Milk Quality Traits

Table 2 summarizes the effects of diet on the daily and total yields of milk, fat, protein, lactose, total solids, logSCC, and logTBC, as well as on pH, EC, and RI. A significant effect was observed on the daily and total fat yields (*p* = 0.032 and *p* = 0.037, respectively) and on EC (*p* < 0.001). Namely, ewes in group E yielded more fat in their milk (daily ca. 8.6 g, 95% CI, 0.7 to 16.4 g, and total ca. 800 g, 95% CI 50.4 to 1549.57 g) compared to ewes in group C. Furthermore, milk EC was decreased by 0.2 mS/cm (*p* < 0.001, 95% CI, −0.3 to −0.1 mS/cm) in group E ewes.

### 3.3. The Effects of Other Explanatory Variables on the Daily and Total Milk Yields and Milk Quality Traits

The sampling occasion had a statistically significant effect (*p* < 0.001) in every case, except from the RI and the logarithm of TBC. Moreover, Farm A ewes had significantly higher logarithm of SCC and TBC (*p* < 0.001, increased by 0.44 and 0.30 logarithms, respectively) and an increased milk EC by 0.48 mS/cm (*p* < 0.001) compared to Farm B. The parity number had no statistically significant effect on any of the studied traits, while a one-degree increase on BCS was associated with a decrease in pH by 0.08 units (*p* < 0.01) and milk EC by 0.27 mS/cm. Regarding the total milk yield and the quality traits (fat, protein, lactose, and total solids yield), the farm and parity number had no statistically significant effects on any case, except from Farm B ewes that had a significantly higher total fat yield compared to Farm A (*p* < 0.05, increased by 1022.6 g).

## 4. Discussion

To the best of our knowledge, this is the first time the effects of SBM substitution with a mixture of rapeseed meal, cottonseed cake, and fava beans on milk performance in dairy sheep have been prospectively studied; no significant effects were observed on MY and MQT, with the exception of a favorable effect on the milk fat yield observed in the experimental ration. The combination of alternative protein sources aims towards the efficient coverage of the metabolizable protein requirements of dairy sheep through the improvement of the rumen degradable protein (RDP)-to-rumen undegradable protein (RUP) ratio and the balance of essential amino acids [15,30]. As the amino acid profile differs significantly among the protein sources, the ideal diet should be formulated by a panel of protein sources which complement microbial proteins with the essential amino acids for milk production, such as methionine, lysine, leucine, and histidine [31].

The majority of relevant studies in the available literature have assessed the effects of substituting SBM with a single alternative protein feed, mainly on the quantity and quality of milk in dairy cows. Therefore, extrapolating and directly comparing the results of these studies with our findings is not appropriate.

The components selected in the experimental ration, namely, rapeseed meal, cottonseed cake, and fava beans, are among the most commonly used alternative protein sources for the substitution of SBM in small ruminant diets in Greece, as the crops they derive from are popular in different regions around the country. However, the effectiveness of integrating them into the dairy sheep diets to effectively substitute SBM has not been assessed, until now, on an evidential basis and under commercial farming conditions. Up-to-date data demonstrating the effects of SBM replacement with rapeseed meal on MY and MQT in dairy sheep are scarce. On the contrary, relevant studies in dairy cows have extensively documented these effects and rapeseed meal was found to be more effective than SBM and other oilseed feeds, favoring their milk, protein, lactose, and fat yields without affecting BCS [31,32,33,34,35]. The decrease in milk urea and urine urea nitrogen, and the increase of the essential amino acid availability, such as histidine, methionine, and lysine, in cows fed rapeseed meal, restates SBM superiority in terms of ruminal degradability, protein digestibility, and nitrogen efficiency [32,34,35,36].

Cottonseed cakes have been exploited as an alternative to the SBM protein source mainly in monogastric farm animals, for fattening lambs, and for growing goats, and less commonly in adult ruminants. Therefore, data with which to compare the results of our study are limited. The addition of cottonseed cake in ostriches and broilers improved their growth rate [10,22], whereas in pigs, the lysine deficiency and the presence of gossypol adversely affected their performance [37]. The substitution of SBM with cottonseed cake in goat kids and lambs did not influence their growth or their feeding efficiency, as well as the microbial protein synthesis [16,38].

The substitution of SBM with fava beans has been studied in dairy ewes in Italy [39,40]. Liponi et al. [39] replaced soybean meal with fava beans or peas in 18 postweaning Massese-bred lactating ewes for 70 days, while Bonanno et al. [40] replaced maize grain and SBM with fava beans, chickpeas, or peas mixed with barley in 12 Comissana-bred lactating ewes for 21 days. In both cases, no statistically significant differences regarding MY, MQT, BCS, SCC, TBC, and pH were observed. On the contrary, in goats, fava beans improved the milk protein yield when compared to other feeds with a high protein content (e.g., sunflower meal, vetch, and bitter vetch) [41,42]. Relevant studies in dairy cows indicated that the partial [43] or total replacement of SBM with fava beans [44] or with a combination of fava beans and rapeseed meal [26] did not adversely affect the milk yield and composition, which is in accordance with our findings. However, the combination of fava beans with peas reduced the dry matter intake and milk yield in cows [12].

In the current study, daily and total milk fat yields were significantly increased (by ca. 12.0%) in the group E, compared to the group C, ewes. An obvious explanation could be the increased content of NDF against the starch content in the experimental ration, as previously reported in dairy sheep and cows [45,46,47,48]. Although the mechanisms and regulators of the milk fat synthesis are quite complicated and insufficiently evidenced in dairy sheep, high starch and low NDF content have been related to the disruption of the acetate-to-propionate and acetate-to-butyrate ratios, with a further effect on rumen fermentation and the production of fatty acids for milk fat synthesis [49,50]. In any case, other factors affecting the efficiency of the experimental rations need to be further elucidated and more studies under different experimental protocols are warranted to reveal potential differences on the ruminal metabolism and feed digestibility of the two rations, and to evidence the superiority claims of the experimental rations.

In our study, the mean values of BCS and their progress were similar in the two groups and followed the expected pattern for the studied period (mid to late lactation) [51]. The two diets succeeded in meeting the energy demands of the milking ewes without compromising their energy balance during the mid to late stages of lactation. Although high starch diets favor fat deposition against milk production, causing an increase of BCS in mid-lactating animals [46,52,53], this was not the case in our study. A possible explanation could be the regular modification of feedstuff quantities to efficiently meet, but not exceed, the nutritional demands of the ewes according to the stage of lactation and productivity.

The studied milk physical properties did not differ between the two groups, except with EC, which was significantly lower in group E. Although an increased EC has been associated with subclinical mastitis and increased SCC in dairy cows and small ruminants [52,53], in our study, we cannot conclude the potential improved mammary gland health status in the group E ewes, since SCC and TBC values were not significantly lower in that group. Moreover, EC is affected by the milk chemical composition, among other physiological factors, such as the parity number, lactation stage, and animal breed [54]. Specifically, it is negatively correlated with the milk fat content in dairy cows and small ruminants due to the nonconductive properties of fat globules [54,55], possibly explaining this decrease in the milk EC in the group E ewes, which presented significantly increased daily and total milk fat yields compared to the group C ewes. Nevertheless, this is an assumption which needs to be further Investigated to reveal the underlying mechanisms justifying the variation of milk EC. For this reason, a larger-scale assessment of health indicators of sheep fed with the two diets is necessary to conclude the effects of the two studied diets on the animal health and milk hygiene status.

Despite the encouraging results regarding the use of alternative protein sources, the gradual replacement of SBM in the diets of dairy sheep prerequisites addressing some limiting factors. In Greece and in many other European countries, the lack of experience and expertise in the cultivation of alternative protein crops (fava beans, rapeseed meal, lupin etc.) results in a low crop performance and the inadequate standardization of qualitative traits (protein, fat, moisture content, foreign material, etc.). In our study, the cost of the experimental ration was from EU 1.0 to EU 1.5/kg less than the control ration, despite the observed shortage of fava bean and the consequent increase in its price in the year of the study. In any case, the large-scale use of alternative protein-rich feedstuffs requires the extended cultivation of the respective crops to maintain a competitive cost against conventional rations (with SBM) and to succeed in sustainable production.

## 5. Conclusions

The partial replacement of SBM, used as the main protein source in a typical commercial concentrate ration for dairy sheep, with a mixture of rapeseed meal, cottonseed cake, and fava beans did not adversely affect the milk yield and any of the studied milk quality traits; on the contrary, it was associated with a favorable effect on daily and 100-day fat yields and on milk EC. The increase in the milk fat yield of ewes fed the experimental ration is possibly related to its greater NDF content. On the other hand, the decrease of EC in the experimental ration group may be linked to the increased milk fat content. However, the potential mechanisms justifying these findings need to be further investigated, assessing, at the same time, the nitrogen utilization, nutrient digestibility, and ruminal metabolism of the control and experimental rations. Although the results of our study support the efficient use of alternative protein sources for the substitution of SBM in the diets of intensively reared dairy sheep, further studies are deemed crucial for the assessment of the total replacement of SBM in terms of the animal health and productivity statuses and the overall farm sustainability.

## Figures and Tables

**Table 1:** Composition and chemical analysis of the rations fed in the control (C) and experimental (E) groups.

	Control Ration	Experimental Ration
**Composition (%)**		
Soybean meal	20.0	5.0
Rapeseed meal	-	13.0
Cottonseed cake	-	10.0
Fava beans	-	12.5
Barley grain	13.5	-
Corn grain	50.0	52.0
Wheat bran	14.0	-
Sugar beet pulp	-	5.0
Vitamins and minerals	2.5	2.5
**Chemical analysis**		
Dry matter (%)	86.73	88.18
Crude protein (% of DM)	15.63	15.96
Ash (% of DM)	4.32	4.91
Fat (% of DM)	3.19	3.51
NDF (% of DM)	8.00	13.45
ADF (% of DM)	1.76	6.74
ADL (% of DM)	0.04	1.86
Starch (% of DM)	50.63	39.82
Calcium (% of DM)	1.61	1.71
Phosphorus (% of DM)	0.52	0.45
Net energy for lactation ^†^ (Mcal per kg DM)	1.95	1.96

DM: dry matter; NDF: neutral detergent fiber; ADF: acid detergent fiber; ADL: acid detergent lignin; ^†^ theoretical estimation using the software Plurimix System^®^ v.2.41.34, (Fabermatica, Ostiano, Italy).

**Table 2:** Mean values (± SD) of daily and total milk yields and milk quality traits and the effects of diet on them (reference category for comparisons is group C).

							95% CI	
	Dependent Variables	Group C Mean (± SE)	Group E Mean (± SE)	*Β*	SEM	*p*-Value	Lower Bound	Upper Bound
Model (1)	Daily milk yield (L)	1.09 (0.28)	1.09 (0.28)	0.01	0.081	0.946	−0.15	0.14
	Daily fat yield (g)	67.64 (18.46)	76.20 (18.44)	8.55	3.945	0.032	0.73	16.38
	Daily protein yield (g)	62.30 (20.61)	66.11 (20.58)	3.81	4.219	0.369	−12.17	4.56
	Daily lactose yield (g)	51.30 (59.3)	52.44 (59.52)	1.14	3.686	0.757	−8.45	6.16
	Daily total solids yield (g)	189.64 (63.53)	205.04 (63.78)	15.40	12.020	0.203	−39.23	8.44
	Log of SCC (10^3^/mL)	5.68 (0.45)	5.70 (0.45)	−0.02	0.092	0.824	−0.02	0.16
	Log of TBC (cfu × 10^3^/mL)	4.50 (0.27)	4.49 (0.27)	−0.02	0.066	0.783	−0.11	0.15
	pH ^†^	6.60 (0.09)	6.57 (0.09)	−0.04	0.021	0.068	0.00	0.08
	Electrical conductivity (mS/cm) ^†^	3.58 (0.08)	3.39 (0.08)	−0.20	0.054	0.000	−0.31	−0.09
	Refractive index ^†^ (brix)	15.38 (0.57)	15.25 (0.61)	−0.12	0.685	0.858	−1.24	1.48
Model (2)	100-day milk yield (L)	109.57 (±5.45)	109.26 (±4.68)	−1.10	7.216	0.879	−15.41	13.21
	100-day fat yield (g)	6863.06 (±266.76)	7728.33 (±274.00)	799.96	378.095	0.037	50.35	1549.57
	100-day protein yield (g)	6161.33 (±297.23)	6508.12 (±275.18)	281.09	410.284	0.495	−532.34	1094.52
	100-day lactose yield (g)	5070.31 (±268.36)	5124.32 (±230.58)	4.47	355.361	0.990	−700.66	709.61
	100-day total solids yield (g)	18953.30 (±842.55)	20410.30 (±791.83)	1261.97	1161.835	0.280	−1041.48	3565.42

Group C: control group; Group E: experimental group; SE: standard error; B: coefficient; SEM: standard error of the mean of B coefficient; model (1): mixed linear regression model for daily milk yield and milk quality traits; model (2): linear regression model for 100-day milk yield and milk quality traits; SCC: somatic cell count; TBC: total bacterial count; cfu: colony-forming unit; ^†^ measured at 20 °C.

## Data Availability

Not applicable.
